# Supplementary material for: A Critical View of the Application of the APEX Software (Aqueous Photochemistry of Environmentally-Occurring Xenobiotics) to Predict Photoreaction Kinetics in Surface Freshwaters
Source: Molecules. 2019 Dec 18;25(1):9. doi: 10.3390/molecules25010009 (PMC7017383; doi:10.3390/molecules25010009)
Supplement: Supplementary file 1 [file molecules-25-00009-s001.zip › 3.2.4_gcc-4.4.0.zip!/3.2.4_gcc-4.4.0/3.2.4_gcc-4.4.0/doc/octave/html/interpreter/Persistent-Variables.html]

Persistent Variables - Untitled


Next: Status of Variables,
Previous: Global Variables,
Up: Variables


---

### 7.2 Persistent Variables

A variable that has been declared persistent within a function
will retain its contents in memory between subsequent calls to the
same function. The difference between persistent variables and global
variables is that persistent variables are local in scope to a
particular function and are not visible elsewhere.

The following example uses a persistent variable to create a function
that prints the number of times it has been called.

```
     function count_calls ()
       persistent calls = 0;
       printf ("'count_calls' has been called %d times\n",
               ++calls);
     endfunction
     
     for i = 1:3
       count_calls ();
     endfor
     
     -| 'count_calls' has been called 1 times
     -| 'count_calls' has been called 2 times
     -| 'count_calls' has been called 3 times
```

As the example shows, a variable may be declared persistent using a
`persistent` declaration statement. The following statements are
all persistent declarations.

```
     persistent a
     persistent a b
     persistent c = 2
     persistent d = 3 e f = 5
```

The behavior of persistent variables is equivalent to the behavior of
static variables in C. The command `static` in Octave is also
recognized and is equivalent to `persistent`.

Like global variables, a persistent variable may only be initialized once.
For example, after executing the following code

```
     persistent pvar = 1
     persistent pvar = 2
```

the value of the persistent variable `pvar` is 1, not 2.

If a persistent variable is declared but not initialized to a specific
value, it will contain an empty matrix. So, it is also possible to
initialize a persistent variable by checking whether it is empty, as the
following example illustrates.

```
     function count_calls ()
       persistent calls;
       if (isempty (calls))
         calls = 0;
       endif
       printf ("'count_calls' has been called %d times\n",
               ++calls);
     endfunction
```

This implementation behaves in exactly the same way as the previous
implementation of `count_calls`.

The value of a persistent variable is kept in memory until it is
explicitly cleared. Assuming that the implementation of `count_calls`
is saved on disk, we get the following behavior.

```
     for i = 1:2
       count_calls ();
     endfor
     -| 'count_calls' has been called 1 times
     -| 'count_calls' has been called 2 times
     
     clear
     for i = 1:2
       count_calls();
     endfor
     -| 'count_calls' has been called 3 times
     -| 'count_calls' has been called 4 times
     
     clear all
     for i = 1:2
       count_calls();
     endfor
     -| 'count_calls' has been called 1 times
     -| 'count_calls' has been called 2 times
     
     clear count_calls
     for i = 1:2
       count_calls();
     endfor
     -| 'count_calls' has been called 1 times
     -| 'count_calls' has been called 2 times
```

That is, the persistent variable is only removed from memory when the
function containing the variable is removed. Note that if the function
definition is typed directly into the Octave prompt, the persistent
variable will be cleared by a simple `clear` command as the entire
function definition will be removed from memory. If you do not want
a persistent variable to be removed from memory even if the function is
cleared, you should use the `mlock` function as described in
See Function Locking.
